# A Comparison of Single Fraction and Multi Fraction Radiosurgery on the Gamma Knife ICON: A Single Institution Review

**DOI:** 10.1016/j.adro.2022.101161

**Published:** 2022-12-28

**Authors:** Joseph P. Loftus, Matthew Shepard, Yun Liang, Alexander Yu, Stephen M. Karlovits, Rodney E. Wegner

**Affiliations:** a Allegheny Health Network Cancer Institute, Division of Radiation Oncology, Monroeville, Pennsylvania; b Allegheny Health Network, Department of Neurosurgery, Monroeville, Pennsylvania

## Abstract

**Purpose:**

Brain metastases are a common development in patients with malignant solid tumors. Stereotactic radiosurgery (SRS) has a long track record of effectively and safely treating these patients, with some limitations to the use of single fraction SRS based on size and volume. In this study, we reviewed outcomes of patients treated using SRS and fractionated SRS (fSRS) to compare predictors and outcomes of those treatments.

**Methods and Materials:**

Two hundred patients treated with SRS or fSRS for intact brain metastases were included. We tabulated baseline characteristics and performed a logistic regression to identify predictors of fSRS. Cox regression was used to identify predictors of survival. Kaplan-Meier analysis was used to calculate survival, local failure, and distant failure rates. A receiver operating characteristic curve was generated to determine timepoint from planning to treatment associated with local failure.

**Results:**

The only predictor of fSRS was tumor volume >2.061 cm^3^. There was no difference in local failure, toxicity, or survival by fractionation of biologically effective dose. Predictors of worse survival were age, extracranial disease, history of whole brain radiation therapy, and volume. Receiver operating characteristic analysis identified 10 days as potential factor in local failure. At 1 year, local control was 96.48 and 76.92% for those patients treated before or after that interval, respectively (*P* = .0005).

**Conclusions:**

Fractionated SRS is a safe and effective alternative for patients with larger volume tumors not suitable for single fraction SRS. Care should be taken to treat these patients expeditiously as a delay was shown to affect local control in this study.

## Introduction

Brain metastases (BMs) are one of the most common complications of malignant solid tumors affecting up to 15 to 20% of adult and 5 to 10% of pediatric oncology patients. Current estimates put the yearly incidence of BMs between 30,000 and 40,000 patients.^1^, ^2^, ^3^ Without treatment, survival remains on the order of weeks to months.^4^, ^5^, ^6^ Treatment options range from surgical resection, whole brain radiation therapy (WBRT), stereotactic radiosurgery (SRS), or some combination of these options. SRS has been a standard treatment option for BMs for >20 years, with recent emergence of fractionated SRS (fSRS) for the treatment of larger BMs, where the balance between safety and efficacy can be quite delicate. Herein, we sought to review the outcomes of patients treated with SRS or fSRS for BMs.

## Methods and Materials

### Patients

The records of 200 patients treated between May 2019 and January 2022 with SRS or fSRS for intact BMs were reviewed. This study was approved through our institutional review board (Study 2019-301).

### Mask immobilization and treatment planning

All patients were treated on the Gamma Knife Icon (Elekta, Stockholm, Sweden), and plans were completed using Gammaplan treatment planning software (version 11.1.1.). All patients (except when noted) had a 1-mm slice thickness, contrast-enhanced, volumetric axial magnetic resonance imaging (MRI) scan ideally obtained within 1 week of SRS for target delineation. Two patients were unable to get an MRI owing to a pacemaker or defibrillator and had a thin slice (1 mm) head computed tomography (CT) with contrast completed in diagnostic radiology for planning purposes. At the time of treatment simulation, all patients had individualized thermoplastic masks and head cushions made on the Gamma Knife Icon. After the mask hardened, a cone beam CT (CBCT) image set with a CT dose index of 6.3 mGy was acquired and used as the stereotactic reference for treatment planning. The patient was then imaged with MRI or diagnostic CT while immobilized with the same mask and cushion system. The planning target volume was the gross target volume with no margin. Dosing and choice of fractionation were picked based on diagnosis, tumor size, and treatment volume as well as on expected treatment time and anticipation of the patient's ability to hold still for an extended time period.^7^^,^^8^ Planning was completed by a physicist in collaboration with a neurosurgeon and radiation oncologist. Planning was typically a combination of inverse and forward planning with a goal of target coverage of 99 to 100%, while limiting normal brain to a V12 of 10 cc for SRS and V24 < 16.8 cc for fSRS.^9^

### Treatment delivery and motion management

On the treatment day, the patient was immobilized with customized mask and head cushion. Right before the treatment, a CBCT with CT dose index of 2.5 mGy was acquired and registered to the initial reference CBCT to identify any spatial shifts. The translational portion of the resulting registration matrix was used to update the short coordinates of the treatment plan. The dose coverage was updated accordingly for evaluation. During the treatment, the high-definition motion monitoring system monitored the reflective nose maker on the patient.

### Statistics

We tabulated baseline characteristics including age, sex, diagnosis, Karnofsky performance status, graded prognostic assessment, the existence of the extracranial disease, past radiation therapy, the number of metastases, tumor volume, treatment dose, treatment fractionation, days from treatment planning to SRS, and survival time from SRS.^1^^,^^3^^,^^5^ We also recorded any distant failures (new BM outside of target volumes) as well as local failures based on Response Assessment in Neuro-Oncology criteria.^10^ Clinical follow-up was defined in months from SRS to most recent clinical visit, and radiologic follow-up was defined in months from SRS to most recent brain imaging. Logistic regression was used to generate odds ratios to identify predictors of fractionation. Cox regression was used to identify predictors of survival. Kaplan-Meier analysis was used to calculate survival, local failure, and distant failure rates. In addition, a receiver operating characteristic curve was generated to determine any potential time point from planning to treatment associated with local failure.

## Results

### Demographics

In Table 1, we detail baseline characteristics of the 200 patients with 681 BMs included in this study. The median age was 62 (range, 29-91) and the median Karnofsky performance status was 80 (range, 50-100). Twenty % of patients had prior WBRT, and 64% had extracranial metastatic disease. Primary malignancies for the cohort were predominantly lung cancer (56%), then melanoma (15%), breast (13%), gastrointestinal (3%), and others (13%). Median target volumes was 0.68 cc for single fraction treatment, 2.585 cc for 3 fractions, and 7.96 cc for 5 fractions.

**Table 1:** Baseline characteristics and odds ratios for likelihood of 1, 3, and 5 factions of stereotactic radiosurgery

Characteristic	All patients (%) (n = 200)	1 fx (%) (n = 63)	3 fx (%) (n = 108)	5 fx (%) (n = 29)	Odds ratio (95% CI)	*P* value
Sex						
Male	81 (41)	27 (43)	44 (41)	10 (34)	Reference	Reference
Female	119 (60)	36 (57)	64 (59)	19 (66)	1.9 (0.84–4.30)	0.1207
Age						
≤61 y	97 (49)	39 (62)	48 (44)	10 (34)	Reference	Reference
>61 y	103 (52)	24 (38)	60 (56)	19 (66)	1.42 (0.59–3.47)	0.4356
Primary malignancy						
Lung (NSCLC/SCLC/LCNEC)	116 (58)	32 (51)	70 (65)	14 (47)	Reference	Reference
Breast	27 (14)	15 (24)	10 (9)	2 (7)	0.49 (0.14–1.73)	0.2702
Melanoma	33 (17)	11 (17)	17 (16)	5 (17)	0.91 (0.34–2.46)	0.8609
GI (anal/colon/rectal)	6 (3)	2 (3)	0 (0)	4 (14)	0.78 (0.10–5.96)	0.8083
Other*	18 (9)	3 (5)	11 (10)	4 (14)	2.28 (0.36–14.55)	0.3839
KPS						
50	3 (2)	1 (2)	2 (2)	0 (0)	Reference	Reference
60	5 (3)	0 (0)	4 (4)	1 (3)	6.60 (0.19–225.81)	0.2951
70	19 (10)	4 (6)	10 (9)	5 (17)	2.21 (0.12–40.33)	0.5918
80	109 (55)	30 (48)	61 (56)	18 (62)	3.21 (0.20–50.52)	0.4072
90	57 (29)	26 (41)	27 (25)	4 (14)	1.60 (0.08–30.89)	0.7572
100	7 (4)	2 (3)	4 (4)	1 (3)	5.59 (0.16–188.13)	0.3374
GPA score						
0-1.0	59 (30)	17 (27)	33 (31)	9 (31)	Reference	Reference
1.5-2.5	117 (59)	35 (56)	63 (58)	19 (66)	1.32 (0.40–4.31)	0.6452
3.0	12 (6)	5 (8)	7 (6)	0 (0)	0.71 (0.07–6.86)	0.7699
3.5-4.0	12 (6)	6 (10)	5 (5)	1 (3)	1.54 (0.15–16.03)	0.7191
Extracranial disease						
No	75 (38)	24 (38)	41 (38)	10 (34)	Reference	Reference
Yes	125 (63)	39 (62)	67 (62)	19 (66)	1.19 (0.41–3.45)	0.7477
Past WBRT						
No	159 (80)	51 (81)	84 (78)	24 (83)	Reference	Reference
Yes	41 (21)	12 (19)	24 (22)	5 (17)	1.60 (0.61–4.17)	0.3380
Total number of metastases						
1	79 (40)	22 (35)	45 (42)	12 (41)	Reference	Reference
2-5	80 (40)	26 (41)	41 (38)	13 (45)	0.87 (0.34–2.25)	0.7754
6-10	28 (14)	9 (14)	16 (15)	3 (10)	0.64 (0.16–2.56)	0.5303
11-15	10 (5)	4 (6)	5 (5)	1 (3)	0.61 (0.1–3.71)	0.5891
17-21	3 (1)	2 (3)	1 (1)	0 (0)	0.46 (0.02–8.66)	0.6055
Total treatment volume (cc)						
0.0048-0.495	50 (25)	25 (40)	22 (20)	3 (10)	Reference	Reference
0.495-2.061	50 (35)	24 (38)	21 (19)	5 (17)	0.74 (0.29–1.90)	0.5343
2.061-7.402	46 (23)	12 (19)	32 (30)	2 (7)	3.93 (1.41–10.94)	0.0087
>7.402	54 (17)	2 (3)	33 (31)	19 (66)	22.79 (4.59–113.21)	0.0001
Total dose						
Minimum	15	15	21	20	-	-
Median	27	21	27	25	-	-
Maximum	30	24	27	30	-	-

*Abbreviations:* CI = confidence interval; fx = faction; GI = gastrointestinal; GPA = graded prognostic assessment; KPS = Karnofsky performance status; LCNEC = large cell neuroendocrine carcinoma; NSCLC = non-small cell lung cancer; SCLC = small cell lung cancer; WBRT = whole brain radiation therapy.

⁎ Prostate, renal cell, thyroid, esophagus, ovarian, bladder.

Almost all patients (99%) had follow-up imaging available for review with a median clinical follow-up of 9 months (range, 0-34) and median imaging follow-up of 6 months (range, 0-31). The median dose was 21 Gy for SRS (range, 15-24), 27 Gy (range, 21-27) for fSRS in 3 fractions, and 25 Gy (range, 20-30) for fSRS in 5 fractions. Logistic regression for the cohorts showed, unsurprisingly, that the only predictor of fSRS over SRS was tumor volume >2.061 cc (*P* = .0087). Serious toxicity was limited in both SRS and fSRS groups, with long-term toxicity ≥grade 3 rates of 0 and 2.5%, respectively.

### Overall survival

**Figure 1:**
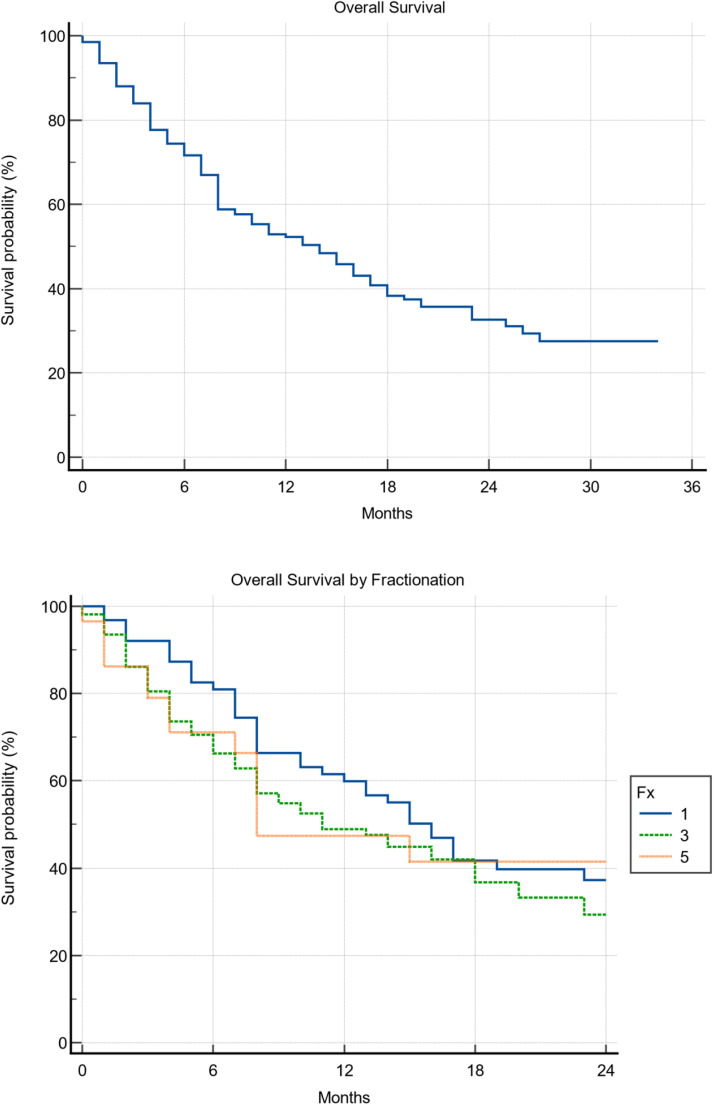
A, Overall survival. The overall survival plot for the full cohort of 200 patients. The median overall survival time was 14 months with a 1-year survival rate of 52%. B, Overall survival by fractionation. This graph displays the overall survival of the cohort be fractionation. The median overall survival was 16, 11, and 8 months for 1, 3, and 5 fractions (fx), respectively (*P* = .52). The corresponding 1-year survival rates were 60, 49, and 47%.

Median survival by fractionation for 1, 3, 5, and overall was 16 months (range, 10-23), 11 months (range, 8-18), 8 months (range, 7-15), and 14 months (range, 10-17), respectively (*P* = .52). Cox regression identified increasing age (*P* = .0106), presence of extracranial disease (*P* = .0052), history of WBRT (*P* = .0003), and increasing tumor volume (*P* = .0419) as predictive of worse survival (Table 2). Of note, fractionation was not found to be a significant predictor in terms of mortality (*P* = .9546; Fig. 1B).

**Table 2:** Univariate Cox regression analysis (survival)

Variable	HR	95% CI	*P* value
Age			
<61	Reference	-	Reference
>61	1.9037	1.1620–3.1190	0.0106
Extra cranial disease			
No	Reference	-	Reference
Yes	2.3537	1.2920–4.2878	0.0052
Stereotactic radiosurgery			
Unfractionated	Reference	-	Reference
Fractionated	0.9863	0.6126–1.5878	0.9546
GPA score			
0-1.0	Reference	-	Reference
1.5-2.5	1.4022	0.7773–2.5296	0.2614
3.0	1.4057	0.3570–5.5348	0.6262
3.5-4.0	1.5885	0.3636–6.9392	0.5384
KPS			
50	Reference	-	Reference
60	2.4299	0.3687–16.0137	0.3561
70	3.4441	0.7229–16.4084	0.1205
80	1.3674	0.2896–6.4558	0.6928
90	1.3791	0.2563–7.4200	0.7081
100	4.4943	0.6828–29.5806	0.1180
Number of metastases			
1	Reference	-	Reference
2-5	1.6842	0.9683–2.9295	0.0649
6-10	1.5613	0.7466–3.2647	0.2365
10-15	2.0934	0.7926–5.5291	0.1360
16-21	0.6711	0.0803–5.6110	0.7128
History of WBRT			
No	Reference	-	Reference
Yes	2.5749	1.5400–4.3054	0.0003
Primary malignancy			
Lung (NSCLC/SCLC/LCNEC)	Reference	-	Reference
Breast	1.0594	0.5400–2.0783	0.8668
Melanoma	1.0410	0.5912–1.8332	0.8893
GI (anal/colon/rectal)	1.1403	0.3889–3.3429	0.8109
Other*	1.0582	0.5134–2.1814	0.8781
Sex			
Male	Reference	-	Reference
Female	0.6631	0.4249–1.0349	0.0705
Tumor volume			
0.0048-0.495	Reference	-	Reference
0.495-2.061	1.3179	0.7264–2.3912	0.3638
2.061-7.402	1.9406	1.0246–3.6756	0.0419
>7.402	1.4349	0.7603–2.7084	0.2652

*Abbreviations:* CI = confidence interval; GI = gastrointestinal; GPA = graded prognostic assessment; HR = hazard ratio; KPS = Karnofsky performance status; LCNEC = large cell neuroendocrine carcinoma; NSCLC = non-small cell lung cancer; SCLC = small cell lung cancer; WBRT = whole brain radiation therapy.

⁎ Prostate, renal cell, thyroid, esophagus, ovarian, bladder.

### Local and distant failure

**Figure 2:**
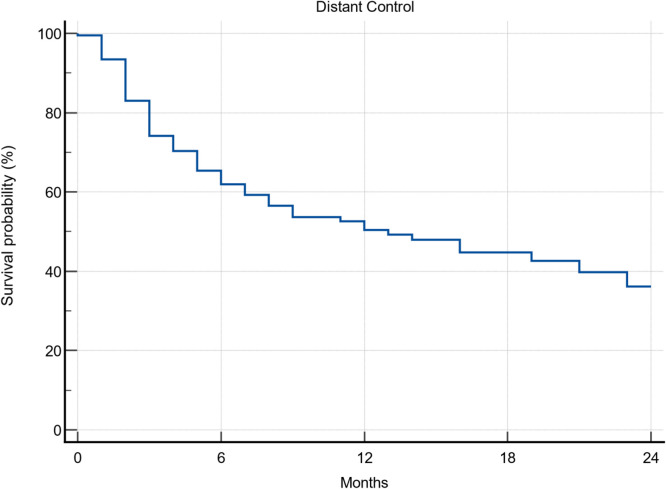
Seventy-eight patients developed distant intracranial failure. The median time to new brain metastases was 13 months, with 38% having new brain metastases at 6 months.

**Figure 3:**
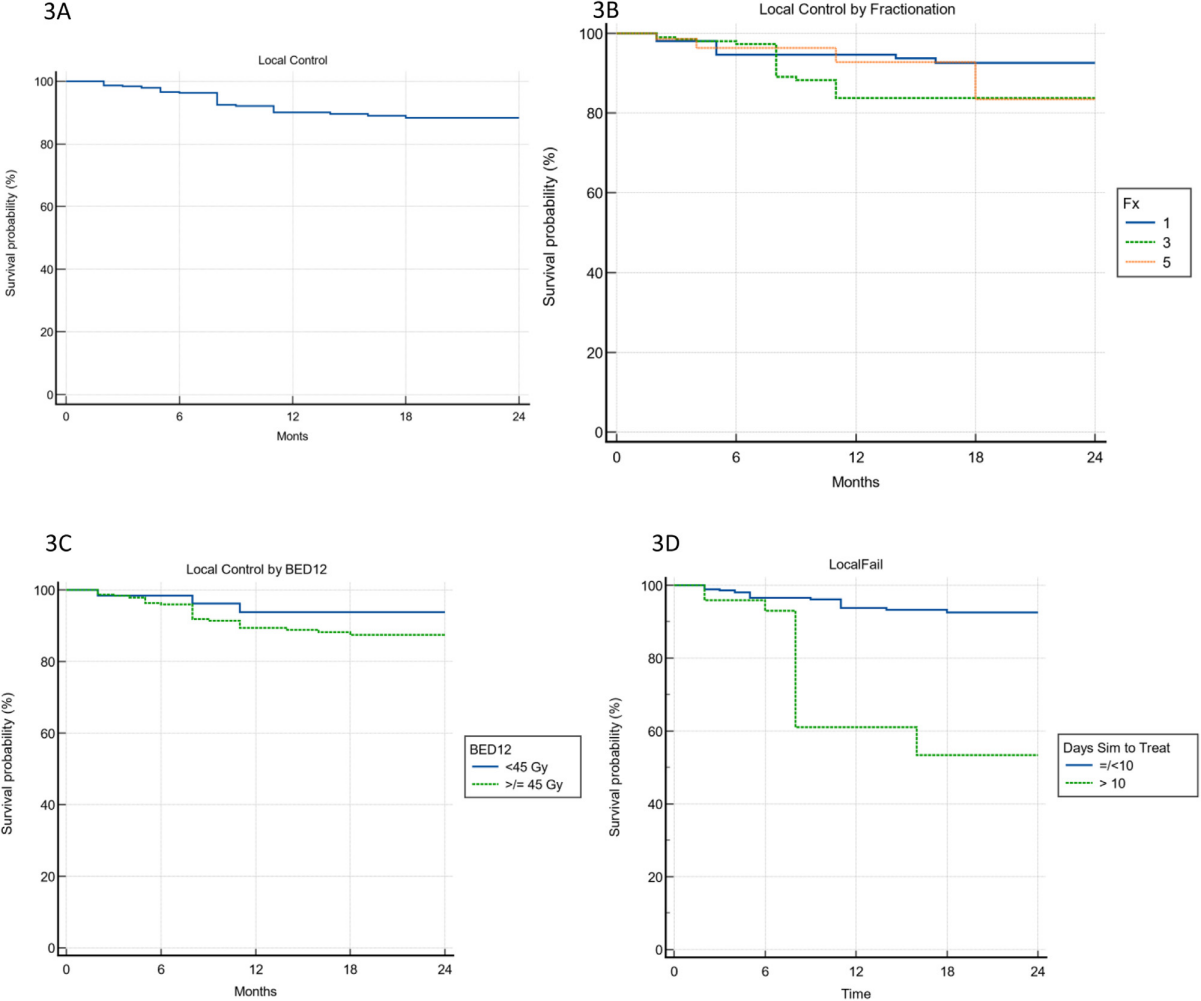
A, Local failure. With follow-up, 38 of the 681 brain metastases failed for a rate of 5.29%. This corresponded to a 1-year local control rate of 90%. B, Local failure by fractionation. The 1-year local control for 1, 3, and 5 fraction stereotactic radiosurgery was 95, 84, and 93%, respectively (*P* = .2675). C, Local failure by biological effective dose. The local control at 1 year for patients with a biological effective dose 12 (BED12) >45 Gy and biological effective dose <45 Gy was 94 and 90%, respectively (*P* = .2795). D, Local failure in days of simulation to treatment. The 1-year local control for patients treated within 10 days of simulation was 94% compared with 61% for those treated greater than 10 days (*P* < .0001). *Abbreviation:* fx = faction; Sim = simulation; Treat = treatment.

In total, 78 patients developed distant brain failure in follow-up. The median time to distant brain failure was 12 months (Fig. 2). For the entire cohort, the 1-year local control was 90%, median not reached (Fig. 3A). There was no significant difference in local failure by fractionation scheme. The 1-year rates of local control were 94.6, 83.8, and 92.8% for 1, 3, and 5 fractions, respectively (*P* = .2675; Fig. 3B). To further compare local control, we calculated local control by biological effective dose (BED) 12 using a cutoff of 45 Gy as described by Minniti et al.^9^ There was again no significant difference in local control (Fig. 3C). Lastly, as described in the methods, we used a receiver operating characteristic analysis to help determine whether there was a timepoint from MRI planning scan to SRS treatment associated with local failure (Fig. E1). The significant figure from that analysis was 10 days. The 1-year local control rates were 93.8 and 61.0% for patients treated within 10 days and after 10 days, respectively (*P* < .0001; Fig. 3D).

## Discussion

The data and results presented here represent one of the largest series of patients treated for BMs in a fractionated manner on the Gamma Knife Icon with a single fraction comparison cohort. Not surprisingly, more patients were treated in a fractionated manner when tumor volume was >2.061 cc; but that should be expected as the purpose of fSRS is to attempt to mitigate toxicity by using fractionation. In that vein, we did find similar, very low levels of serious toxicity in both cohorts (<5%) with no discernable difference. Likewise, there was no significant difference in local control or survival, despite patients in the fSRS group having higher intracranial tumor burden. To investigate the possible causes of local failure we also looked at the BED and time between planning scan and start of treatment. Interestingly, we did not find BED to have significantly contributed to the local failure rate. We did, however, discover that the time between planning and treatment did result in a significantly decreased local control rate with a cutoff of 10 days. This, as expected, suggests that time between planning and treatment implementation is of critical clinical importance.

The use of fractionated SRS has been examined before and reported upon. Minniti et al was 1 of the first groups to report on this subject and reported outcomes in 289 patients with 343 metastases from Rome, Italy.^9^ They found that using a dose of 27 Gy in 3 fractions (40 Gy BED) was more effective than single fraction treatment of 15 to 18 Gy for treating BMs >2 cm. Local control rates were >90% for the fSRS cohort and 77% for single fraction treatment. In addition, the rates of radionecrosis were reduced from 18 to 9%, again in favor of fractionation. The authors did not suggest that this was the optimum dose and recommended future studies were needed to make such a recommendation. However, as seen in our cohort, it appears that 27 Gy in 3 fractions is a standard dose for fSRS. Similar results were seen in a meta-analysis by Lehrer et al, which looked at 24 studies comparing fSRS to single fraction SRS in BMs for patients treated both definitively and in the postoperative setting.^11^ Given the number of included studies, the cohort represented an impressive 1672 patients with 1887 metastases. For patients with BMs 2 to 3 cm in diameter, those treated with fSRS and a BED ranging from 43.2 to 76.2 Gy had a 1-year local control rate of 92.9%; this was in contrast to those patients treated in a single fraction with BED ranging from 32.8 to 65.1 Gy who had a 1-year local control of 77.6% (*P* > 0.05). When looking at outcomes in patients with tumors >3 cm, this difference shrank with 1-year local control of 79.2 and 77.1% for fSRS and SRS, respectively (*P* > 0.05). Interestingly, when SRS was done postoperatively for tumors >3 cm in diameter, the local control rate difference increased to 85.7% for multifractionation (range, 37.9-69.4 Gy BED) and 62.4% for single fractionation (range, 26.4-60 Gy BED), although this was not statistically significant. In terms of radionecrosis, there did appear to be a benefit to fSRS with a reduced rate from 23 to 7% (*P* = .003) for tumors in the 2 to 3 cm range. For tumors >3 cm, the resultant radionecrosis rates were 12 and 7% for SRS and fSRS, respectively (*P* = .29).

In a similar study by Samanci et al from Turkey, fSRS was used in patients with large BMs, defined as volume >4 cm^3^.^12^ The cohort included 58 patients with 79 BMs. They performed fSRS with 2 to 5 fractions and a BED range of 43.2 to 76.2 Gy. With a median follow-up of 1 year, local control was excellent at 96% with no reports of radionecrosis. In addition, the group from University of Pittsburgh Medical Center published on this topic back in 2015.^13^ They reported on outcomes of 36 patients and 37 metastatic lesions treated to slightly lower doses (range, 12-27 Gy; median, 24 Gy). Lesions in that study were on the larger side, median volume 15.6 cm^3^ (range, 10-82.7 cm^3^). In this particular study, local control was lower in comparison to the other studies discussed, with a 1-year local control of 63%. This difference can likely be explained by a combination of the doses used as well as larger treatment volumes.^13^ One of the most recent publications on this topic comes from Sunnybrook and, similar to our series, looked at outcomes in patients with intact BMs treated on the Gamma Knife Icon.^14^ This series include 146 patients with close to 300 BMs. The median prescription dose was 27.5 Gy (range, 20-27.5 Gy) in 5 fractions. With a median follow-up approaching 1 year, the local control was 85%, with notable predictor of local failure being dose ≤25 Gy. Similar to the aforementioned studies, the rate of symptomatic radionecrosis was <5%.

One of the more interesting findings in our study was the association of local failure with increasing time from simulation to actual treatment. Previous studies have investigated the effect of these time lapses leading up to SRS. One of the first studies to highlight this topic was from the University of California San Francisco back in 2015. They reviewed treatment planning schedules and details in 82 patients with 151 BMs, revealing a median time from planning MRI to SRS of 11 days.^15^ Some patients experienced >14 days from MRI to treatment had higher rates of local progression. The 1-year local failure was 25% compared with 66% in favor of interval <14 days. As a follow-up, the same group from the University of California San Francisco compared pretreatment MRI scans with day-of-treatment MRI scans in 165 patients with BMs.^16^ The mean time between these 2 MRI scans was 25 days. The investigators were able to calculate a mean growth rate of 0.02 mL/d across this cohort based on differences in the scans. This corresponded to a 1.35-fold volume increase at 14 days, helping support the results of the prior study. The results seen in our study showing 10 days as the ideal time to treatment falls in line with the aforementioned study conclusions. A small study from France used exponential models to extrapolate tumor volume, as well as time to outgrow target volumes or margins.^17^ The model was developed using 42 patients with melanoma or non-small cell lung cancer and a total of 84 brain scans. Comparing target volumes between diagnostic and planning scans showed an increase in volume for 70 to 96% of patients depending on histology. The shortest time for tumors to expand 1 mm in axial dimension was 6 to 8 days, again depending on histology.

The results of our study certainly fall in line with the aforementioned papers, with a local control rate eclipsing 90% for patients treated <10 days from planning session and low rates of serious toxicity. There are, of course, limitations to our study, the main one being the retrospective nature and inherent selection bias in such a study. Also, this cohort was a heterogeneous population of primary tumors, and 21% had a previous history of WBRT, which could have confounded some of the results. Furthermore, because many patients treated with fSRS have larger tumors, many steroids given around the time of treatment to help reduce potential treatment-related toxicity. Lastly, the median imaging and clinical follow-up were 6 and 9 months, respectively. Longer follow-up would be needed to help confirm the high rates of local control as well as low rates of serious toxicity as radiation necrosis often does not manifest until after 1 year.

## Conclusions

Fractionated SRS is a safe and effective treatment option for patients with larger volume tumors not suitable for single fraction SRS. The excellent local control rates and low toxicity rates seen historically with SRS appear to be preserved. Care should be taken to treat these patients expeditiously as a delay was shown to affect local control in this study.

## Appendix Supplementary materials

Image, application 1
