# Supplementary figures and images for: A Comparison of Single Fraction and Multi Fraction Radiosurgery on the Gamma Knife ICON: A Single Institution Review
Source: Adv Radiat Oncol. 2022 Dec 28;8(2):101161. doi: 10.1016/j.adro.2022.101161 (PMC9943766; doi:10.1016/j.adro.2022.101161)

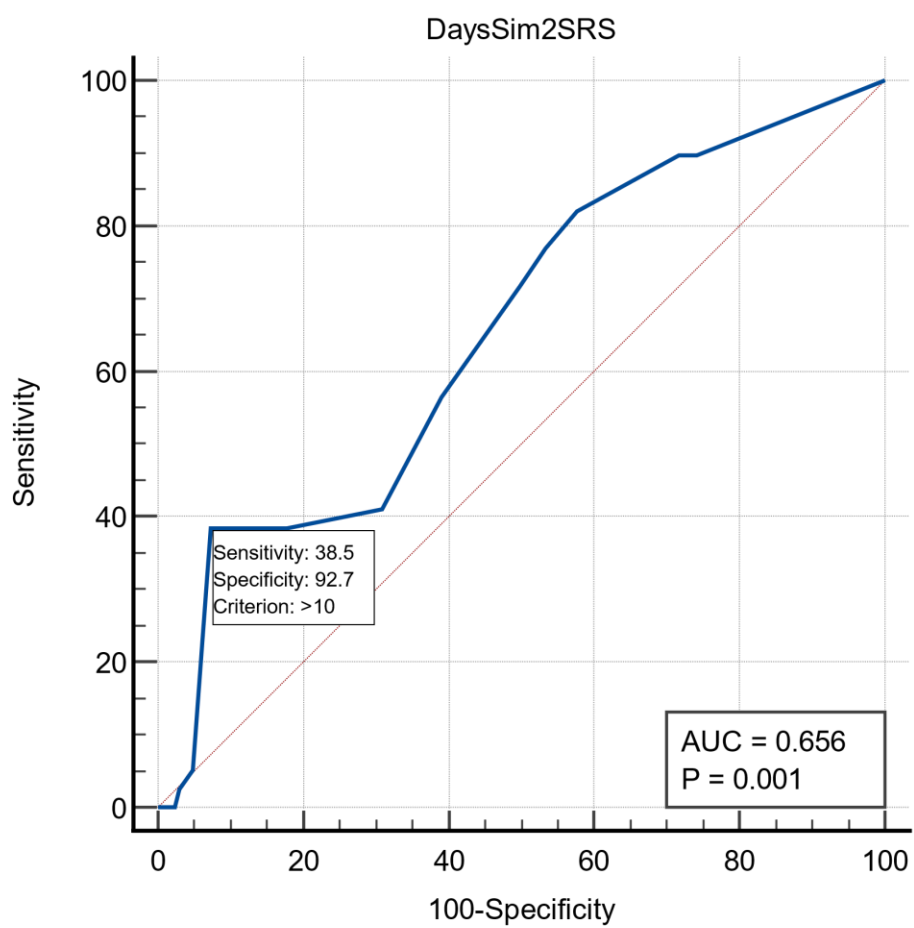

Supplement: Supplementary file 1 [file mmc1.pdf]
